# Patient perceptions of anticoagulant treatment with dabigatran or a vitamin K antagonist for stroke prevention in atrial fibrillation according to region and age: an exploratory analysis from the RE-SONANCE study

**DOI:** 10.1007/s11239-021-02450-2

**Published:** 2021-04-30

**Authors:** Dragos Vinereanu, Dmitry Napalkov, Jutta Bergler-Klein, Bela Benczur, Martin Ciernik, Nina Gotcheva, Alexey Medvedchikov, Pentti Põder, Dragan Simić, Andris Skride, Wenbo Tang, Maria Trusz-Gluza, Jiří Vesely

**Affiliations:** 1 University of Medicine and Pharmacy Carol Davila, University and Emergency Hospital, Bucharest, Splaiul Independentei 169, 050078 Bucharest, Romania; 2 I.M. Sechenov First Moscow State Medical University (Sechenov University), Moscow, Russian Federation; 3 Department of Cardiology, University Clinic of Internal Medicine II, Medical University of Vienna, Vienna, Austria; 4 Balassa Janos County Hospital, Szekszárd, Hungary; 5 Boehringer Ingelheim RCV GmbH & Co. KG, Vienna, Austria; 6 National Cardiology Hospital, Sofia, Bulgaria; 7 North Estonia Medical Centre Foundation, Tallinn, Estonia; 8 Clinic of Cardiology, Clinical Centre of Serbia, Faculty of Medicine, University of Belgrade, Belgrade, Serbia; 9 Pauls Stradins Clinical University Hospital, Riga Stradinš University, Riga, Latvia; 10 Boehringer Ingelheim Pharmaceuticals, Inc, Ridgefield, CT USA; 11 Silesian Medical University, Katowice, Poland; 12 Faculty of Medicine in Hradec Kralove, Charles University and Edumed s.r.o, Broumov, Czech Republic

**Keywords:** Atrial fibrillation, Dabigatran, Non‐vitamin K antagonist oral anticoagulant, Patient perception, Stroke prevention, Warfarin

## Abstract

**Background:**

The oral anticoagulant dabigatran offers an effective alternative to vitamin K antagonists (VKAs) for stroke prevention in atrial fibrillation (AF), yet patient preference data are limited. The prospective observational RE-SONANCE study demonstrated that patients with AF, newly initiated on dabigatran, or switching to dabigatran from long-term VKA therapy, reported improved treatment convenience and satisfaction compared with VKA therapy. This pre-specified sub-study aimed to assess the impact of country and age on patients’ perceptions of dabigatran or VKA therapy in AF.

**Methods:**

RE-SONANCE was an observational, prospective, multi-national study (NCT02684981) that assessed treatment satisfaction and convenience in patients switching from VKAs to dabigatran (Cohort A), or newly diagnosed with AF receiving dabigatran or VKAs (Cohort B), using the PACT-Q questionnaire. Pre-specified exploratory outcomes: variation in PACT-Q2 scores by country and age (< 65, 65 to < 75, ≥ 75 years) (both cohorts); variation in PACT-Q1 responses at baseline by country and age (Cohort B).

**Results:**

Patients from 12 countries (Europe/Israel) were enrolled in Cohort A (*n* = 4103) or B (*n* = 5369). In Cohort A, mean (standard deviation) PACT-Q2 score increase was highest in Romania (convenience: 29.6 [23.6]) and Hungary (satisfaction: 26.0 [21.4]) (*p* < 0.001). In Cohort B, mean (standard error) increase in PACT-Q2 scores between dabigatran and VKAs was highest in Romania (visit 3: 29.0 [1.3]; 24.5 [0.9], *p* < 0.001). Mean PACT-Q2 score increase by age (all *p* < 0.001) was similar across ages. PACT-Q1 responses revealed lowest expectations of treatment success in Romania and greatest concerns about payment in Estonia, Latvia, and Romania, but were similar across ages.

**Conclusions:**

Treatment satisfaction and convenience tended to favor dabigatran over VKAs. Regional differences in treatment expectations exist across Europe.

**Trial and clinical registry:**

Trial registration number: ClinicalTrials.gov NCT02684981.

Trial registration date: February 18, 2016.

**Supplementary Information:**

The online version contains supplementary material available at 10.1007/s11239-021-02450-2.

## Highlights

- This was an exploratory analysis of the RE-SONANCE study.
- AF patients from Europe and Israel prescribed dabigatran or VKA were included.
- Perceptions of anticoagulant treatment were assessed by country and age via PACT-Q.
- Treatment satisfaction and convenience tended to favor dabigatran over VKAs.
- Regional differences in treatment expectations in AF exist across Europe.

## Introduction

Current treatment guidelines recommend oral anticoagulant (OAC) therapy for patients with atrial fibrillation (AF) at risk of thromboembolic events or stroke [1, 2]. For many years, vitamin K antagonists (VKAs), such as warfarin, have been the only available OAC therapy for patients with AF who are at risk of stroke. However, their narrow therapeutic window, need for dose adjustment and monitoring, risk of intracranial hemorrhage, and multiple interactions with other drugs and food make VKAs difficult to manage in routine clinical practice, resulting in underuse and suboptimal adherence [3–5]. A number of non-VKA oral anticoagulants (NOACs) have since been introduced as an alternative to VKA therapy.

NOAC treatment, including the direct thrombin inhibitor dabigatran, in patients with AF who are indicated for anticoagulation treatment has shown to be just as effective as VKA treatment in reducing the risk of stroke (dabigatran 150 mg twice daily is associated with lower rates of stroke and systemic embolism compared with warfarin) [6]. Importantly, NOACs are not associated with the same pharmacological issues as VKAs that affect adherence.

Current guidelines for stroke prevention recommend the involvement of patients in treatment decisions [1, 2, 7], and patient preference may be key in adherence to long-term anticoagulant therapy. Previous studies have reported mixed outcomes regarding quality of life (QoL) and patient preferences for NOAC and VKA therapy for stroke prevention in AF [8–11]. Nevertheless, there remain limited data on patient preference regarding NOAC and VKA therapy, particularly in terms of country- or age-specific preferences.

The prospective observational RE-SONANCE study, conducted using the validated Perception of Anticoagulant Treatment Questionnaire (PACT-Q), demonstrated that patients with non-valvular AF, newly initiated on dabigatran, or switching to dabigatran from long-term VKA therapy, reported improved treatment convenience and satisfaction compared with VKA therapy [12]. In this pre-specified, exploratory analysis from the RE-SONANCE study, variations in treatment convenience and satisfaction (PACT-Q2) scores between countries and age groups were examined. Variations in treatment expectation (PACT-Q1) scores at baseline between countries and age groups were also explored.

## Methods

### Patients and study design

Methods for the RE-SONANCE study have been described previously [12]. In brief, RE-SONANCE was an observational, multi-national study (NCT02684981) conducted in Austria, Bulgaria, the Czech Republic, Estonia, Hungary, Israel, Latvia, Poland, Romania, Russia, Serbia, and Slovenia. The study included physiologists who worked at sites that reflected clinical practice in their country and regularly prescribed dabigatran and VKA for stroke prevention in patients with AF. Patients enrolled were at least 18 years old with non-valvular AF and an indication for anticoagulation therapy for stroke prevention, and not currently participating in any clinical trial or registry. Patients were assigned to 1 of 2 cohorts: Cohort A, which comprised patients with non-valvular AF who were switched from a VKA to dabigatran based on the product label and at the physician’s discretion (≥ 3 months’ continuous VKA treatment for stroke prevention prior to baseline); or Cohort B, which comprised patients newly diagnosed with AF initiated on either dabigatran or VKA (without use of any OAC within 1 year before enrollment). This study was carried out in accordance with the Declaration of Helsinki, International Conference of Harmonisation Tripartite Guideline, Good Clinical Practice, Guidelines for Good Epidemiological Practice and Good Pharmacoepidemiology Practice. The study was initiated in centers once approved by the respective Institutional Review Board/Independent Ethics Committee and competent authority, according to national and international regulations.

### Assessments and outcomes

Patient characteristics and treatment data were collected during routine clinic visits (V) over an observation period of approximately 6 months, at three recommended time points: V1 (baseline; switch from VKA to dabigatran, or initiation of dabigatran/VKA), V2 (30–45 days after baseline), and V3 (150–210 days after baseline). However, if visits occurred outside these time periods, data were collected and assigned to visits based on: V2, 7–124 days after baseline; V3, 125–356 days after baseline. Patients completed the self-administered PACT-Q face-to-face in the clinic [13, 14].

PACT-Q1 assessed patient expectations of anticoagulation therapy, and was administered at V1 for Cohort B only. PACT-Q2 assessed convenience, burden of disease and treatment, and anticoagulant treatment satisfaction; it was administered at V1 (Cohort A only), V2, and V3 (Cohorts A and B). For more information on PACT-Q, refer to Vinereanu et al. [12].

Pre-specified exploratory outcomes included the variation of PACT-Q2 scores across different countries, and in different age groups (< 65, 65 to < 75, and ≥ 75 years) (Cohorts A and B); and variation in PACT-Q1 scores at V1 in different age groups, and geographical variation in treatment perception (Cohort B).

### Statistical methods

The enrolled population comprised all patients who fulfilled the eligibility criteria. The main analysis set (MAS) comprised all eligible patients with known treatment. The propensity score matched set in Cohort B comprised all patients matched with a 1:*n* ratio (VKA:dabigatran) based on propensity scores calculated using a logistic regression model. Summary statistics were prepared for demographic and baseline characteristics, healthcare system characteristics, and physician-rated risk scores, based on the MAS [12].

#### Assessment of PACT-Q2 scores

For Cohort A, mean differences in PACT-Q2 scores between visits were assessed using paired *t*-tests or signed-rank Wilcoxon test. For Cohort B, mean differences in PACT-Q2 scores between treatment subgroups were assessed using propensity score matched analysis. The baseline variables used in the propensity score matched analysis included sex (male/female); age (< 65, ≥ 65 to < 75, ≥ 75 years); status of reimbursement for anticoagulation therapy (reimbursed, partially reimbursed, private pay, other); specialty of the treating physician (cardiologist, internist, neurologist, general practitioner, other); Hypertension, Abnormal Renal/Liver Function, Stroke, Bleeding History or Pre-disposition, Labile international normalized ratio, Elderly, Drugs/Alcohol Concomitantly (HAS-BLED) score (low risk [< 3], high risk [≥ 3]); CHA_2_DS_2_-VASc (Congestive heart failure, Hypertension, Age (≥ 75), Diabetes mellitus, Stroke/transient ischemic attack, Vascular disease, Age 65–75, Sex category) score for embolic risk assessment (considered low or intermediate risk if < 2, and high risk if ≥ 2); number of concomitant medications (0, 1–3, ≥ 4); type of concomitant medication (prescription or no prescription of anti-arrhythmics, antiplatelets, or non-steroidal anti-inflammatory drugs); number of concomitant therapies (0, ≥ 1); presence of comorbidities (presence or absence of malignancy, or gastroesophageal reflux disease or gastroduodenal ulcer disease). Due to the variable size of the matched sets, the analysis of PACT-Q2 scores was also based on the random intercept model, where a variance in the unequal groups (1:*n* matching) was compared and one group contained “repeated” observations. For sub-group analysis by country, a minimum sample size of 250 patients for Cohort A and 260 for Cohort B was required. For other sub-groups, a minimum sample size of 100 patients for Cohort A and 200 for Cohort B was required.

#### Assessment of PACT-Q1 scores

For Cohort B, PACT-Q1 scores at baseline were summarized descriptively for all patients and between treatment sub-groups.

## Results

### Patients

A total of 9472 patients with AF were enrolled (enrolled population) from 698 sites in 12 countries (Europe and Israel) to Cohort A (*n* = 4103) or Cohort B (*n* = 5369). The MAS comprised 4100 patients in Cohort A and 5365 in Cohort B (dabigatran, *n* = 3179; VKA, *n* = 2186) [12]. Baseline characteristics are summarized in Table 1. As noted previously, the mean (standard deviation) age of patients at baseline in Cohort A was 70.5 (9.6) years and 68.6 (9.9) years in Cohort B. In general, age groups were evenly distributed between cohorts, with fewer patients aged < 65 years in Cohort A.

A high risk of systemic embolism (CHA_2_DS_2_-VASc score ≥ 2) and a high risk of bleeding complications (HAS-BLED score ≥ 3) were observed in Cohort A (88.3 and 59.2% of patients, respectively) and Cohort B (dabigatran, 87.8 and 29.1% of patients, respectively; VKA, 91.4 and 31.3% of patients, respectively). Most patients had comorbidities and were receiving concomitant medications. Most patients receiving dabigatran in both cohorts received 150 mg twice daily (Table 1). In Cohort A, the mean duration of previous VKA therapy was 34 months (median 19 months).

**Table 1:** Baseline characteristics of patients in the main analysis set

	Cohort A *N* = 4100	Cohort B		
		Dabigatran *N* = 3179	VKA *N* = 2186	Total *N* = 5365
Age, years, mean (SD)	70.5 (9.6)	68.6 (10.1)	68.5 (9.5)	68.6 (9.9)
Age group, n (%)				
< 65 years	1029 (25.1)	1042 (32.8)	723 (33.1)	1765 (32.9)
65 to < 75 years	1552 (37.9)	1154 (36.3)	803 (36.7)	1957 (36.5)
≥ 75 years	1519 (37.0)	983 (30.9)	660 (30.2)	1643 (30.6)
CHA_2_DS_2_-VASc score ≥ 2	3619 (88.3)	2791 (87.8)	1998 (91.4)	4789 (89.3)
HAS-BLED score ≥ 3	2429 (59.2)	925 (29.1)	685 (31.3)	1610 (30.0)
Patients with comorbidities, n (%)	3541 (86.4)	2651 (83.4)	1986 (90.9)	4637 (86.4)
Patients taking concomitant medications, n (%)	3542 (86.4)	2652 (83.4)	1993 (91.2)	4645 (86.6)
Dabigatran dose				
110 mg twice daily	1429 (34.9)	966 (30.4)	–	–
150 mg twice daily	2671 (65.1)	2213 (69.6)	–	–

### Treatment convenience and satisfaction (Cohorts A and B)

#### European cohorts overall

Among the patients switching from a VKA to dabigatran (Cohort A) in the overall analysis set, PACT-Q2 improved significantly for treatment convenience and treatment satisfaction. Mean change for treatment convenience from V1 to V3 was 24.54 (standard deviation [SD] 22.85) and mean change for treatment satisfaction was 21.04 (SD 20.24) (*p* < 0.001). Among the newly initiated patients in Cohort B, PACT-Q2 scores also showed significant improvement for dabigatran versus a VKA (*p* < 0.001). Mean difference in PACT-Q2 scores between dabigatran and VKA at V3 for treatment convenience was 23.34 (standard error [SE] 0.51), and for treatment satisfaction was 19.01 (SE 0.41) [12].

#### By country

**Fig. 1:**
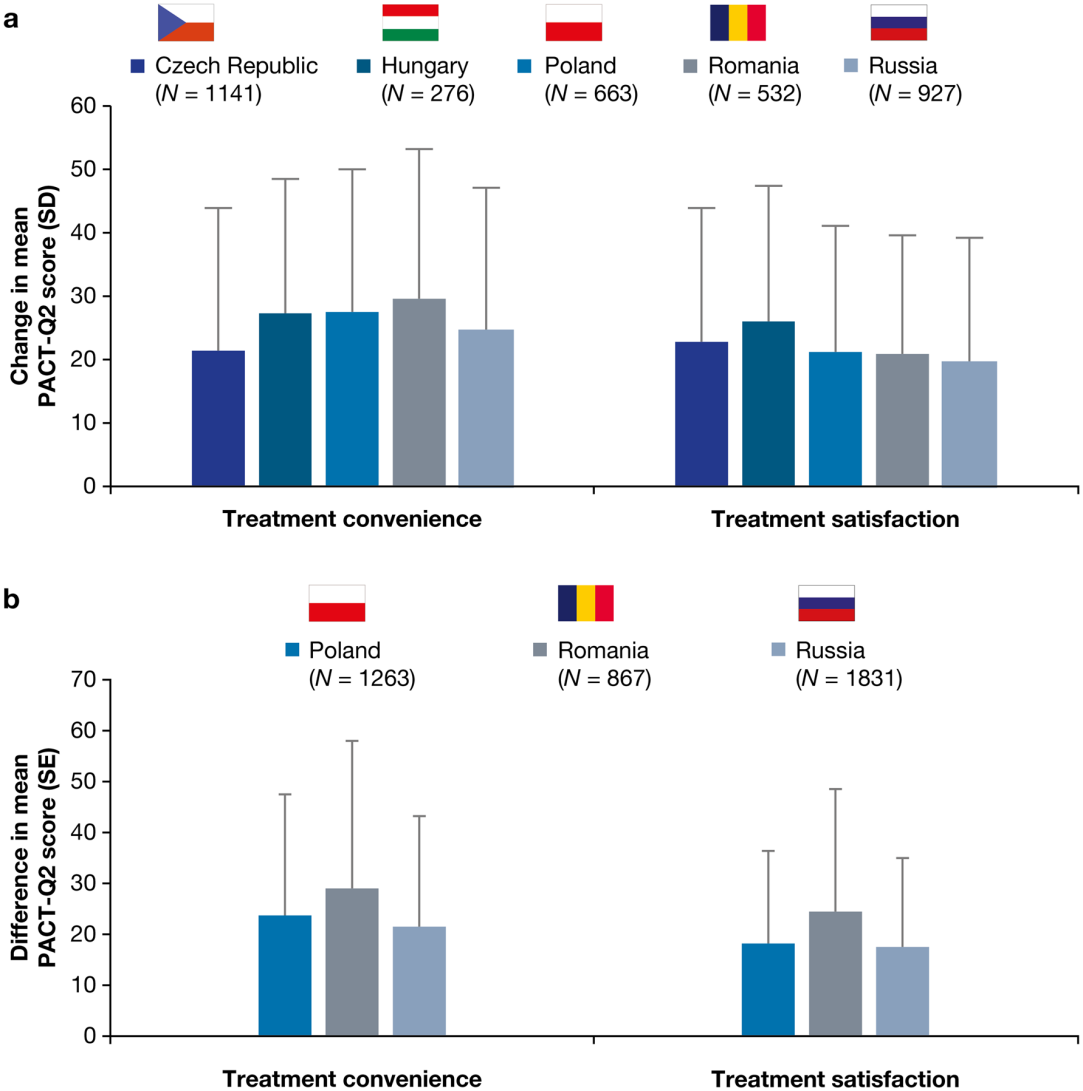
PACT-Q2 score changes in treatment convenience and satisfaction by country (Cohort A, main analysis set; Cohort B, propensity score matched set). **a** Cohort A: patients switched from a VKA to dabigatran; change from V1 to V3. **b** Cohort B: patients newly initiated on dabigatran or a VKA; difference between dabigatran and VKA at V3. Data for the Czech Republic and Hungary are not presented in Fig. 1b because patient numbers meant that changes were statistically inconclusive. All changes were *p* < 0.001 and in favor of the dabigatran sub-group. PACT-Q, Perception of Anticoagulant Treatment Questionnaire; SD, standard deviation; SE, standard error; V1, baseline; V2, initiation period; V3, continuation period; VKA, vitamin K antagonist

Of patients switching from a VKA to dabigatran (Cohort A), a statistically significant mean increase in convenience and treatment satisfaction scores (PACT-Q2 items) between visits was observed only in the Czech Republic, Hungary, Poland, Romania, and Russia (all *p* < 0.001). Changes from V1 to V3 are shown in Fig. 1a. Other countries could not be assessed due to low sample sizes: changes in Austria, Bulgaria, Estonia, Israel, Latvia, Serbia, and Slovenia were statistically inconclusive.

Within Cohort B (newly initiated patients), significant changes in convenience and satisfaction between VKA and dabigatran sub-groups were observed at V2 and V3 for Poland, Romania, and Russia (*p* < 0.001). These changes favored the dabigatran sub-group and tended to be higher at V3 versus V2. Differences between dabigatran and VKA at V3 are shown in Fig. 1b. In some countries, the changes in convenience and satisfaction could not be assessed in Cohort B (Czech Republic and Hungary) or both cohorts (Austria, Bulgaria, Estonia, Israel, Latvia, Serbia, and Slovenia) due to low sample sizes.

#### By age group

**Fig. 2:**
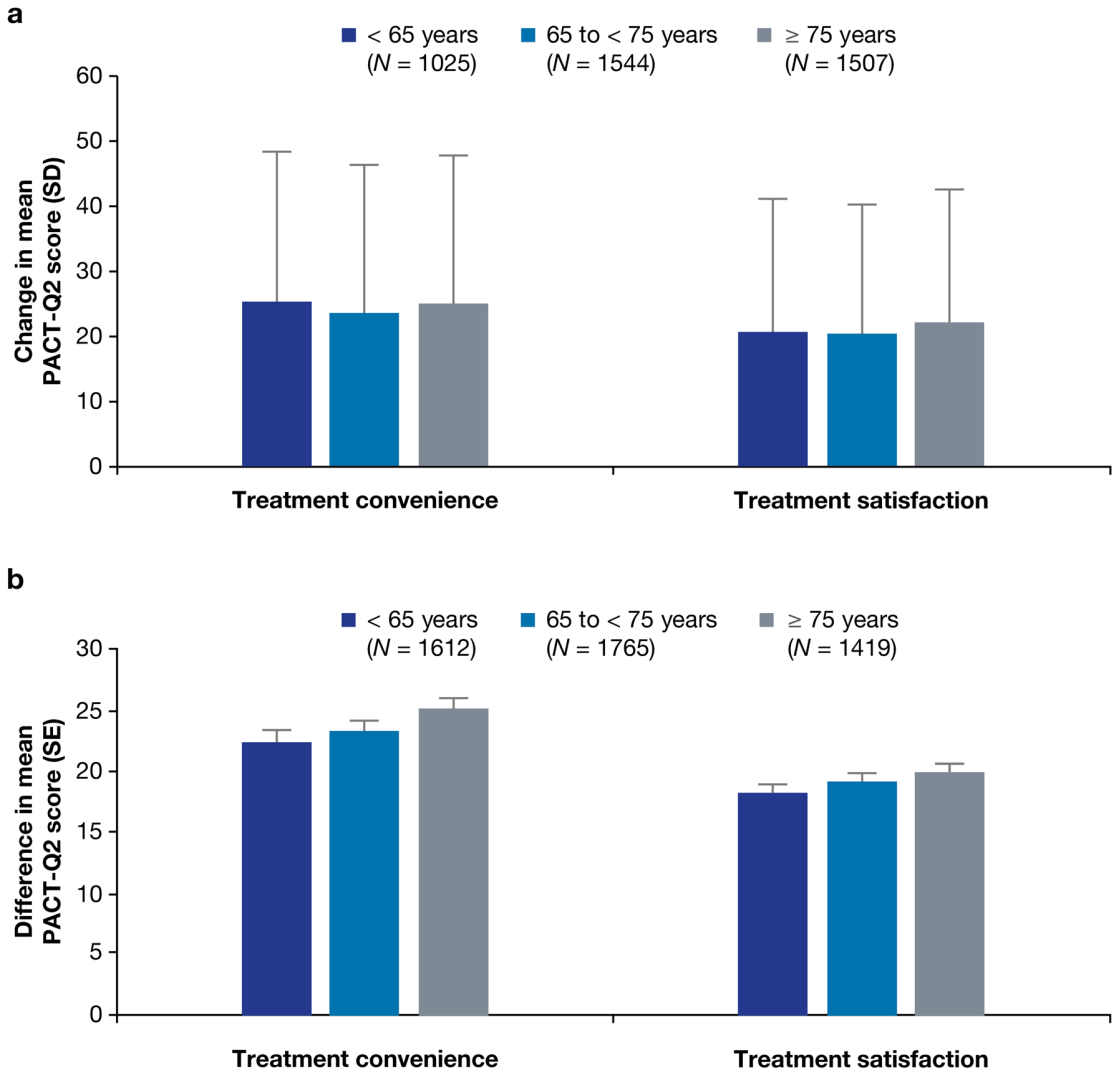
PACT-Q2 score changes in treatment convenience and satisfaction by age group (Cohort A, analysis set; Cohort B, propensity score matched set). **a** Cohort A: patients switched from a VKA to dabigatran; change from V1 to V3. **b** Cohort B: patients newly initiated on dabigatran or a VKA; difference between dabigatran and VKA at V3. All changes were *p* < 0.001 and in favor of the dabigatran sub-group. PACT-Q, Perception of Anticoagulant Treatment Questionnaire; SD, standard deviation; SE, standard error; V1, baseline; V2, initiation period; V3, continuation period; VKA, vitamin K antagonist

Treatment convenience and satisfaction (PACT-Q2 items) among different age groups (< 65, 65 to < 75, ≥ 75 years) in Cohorts A and B are summarized in Fig. 2a and b. Changes in convenience and satisfaction scores between visits (Cohort A) and between dabigatran and VKA sub-groups at V2 and V3 (Cohort B) were generally consistent between age groups. Changes between visits in convenience and satisfaction within all three age groups in both cohorts were statistically significant (*p* < 0.001), and favored the dabigatran sub-group.

### Treatment expectations at baseline (Cohort B)

#### By country

Table 2 summarizes treatment perceptions (PACT-Q1 items) at baseline in patients newly initiated on a VKA or dabigatran (Cohort B) across countries. Low sample sizes meant that some data could not be interpreted (Czech Republic, Hungary, and Slovenia, where *N* < 30) or should be interpreted with caution (Bulgaria, Estonia, Israel, and Latvia, where *N* = 50–100). Overall, most patients were confident that their anticoagulant therapy would prevent blood clots. Most patients in Austria reported this to be either “extremely” (39.8%) or “a lot” (39.5%), while most patients in other countries reported either “moderately” or “a lot.” The expectation that the anticoagulant therapy would provide symptom relief ranged from “a little” to “a lot” for all. Patients in Estonia and Israel had the highest expectations (34.3 and 35.3% reported “a lot,” respectively), while a large proportion of Romanian patients had no expectations (21.5% reported “not at all”). Expectations regarding side effects differed between countries, but with the exception of Austria, Russia, Romania, and Estonia, most patients reported moderate expectations that their anticoagulant therapy would cause side effects such as minor bruises or bleeding. Most patients in all countries considered it important to have an anticoagulant treatment that was easy to take (reporting “a lot” or “extremely”), yet a large proportion of patients in Bulgaria (21.6%), Estonia (17.6%), Israel (29.4%), and Russia (18.3%) found this of only moderate importance. A large proportion of patients in Bulgaria (47.1%) and Israel (41.2%) had moderate concerns about making mistakes when taking their anticoagulant therapy, while Austrian patients reported the least concern (44.2% responded “not at all”). In most countries, a large proportion of patients believed it was important to take care of the anticoagulant therapy themselves (20.8–71.4% responded “a lot”). Notably, a large proportion of patients in Austria (54.4%) and Serbia (42.4%) believed this to be extremely important. Concern about payment for their anticoagulant therapy varied from “moderate” to “a lot” in most countries, with many patients in Estonia (29.4%), Latvia (23.3%), and Romania (26.0%) expressing extreme concern. This contrasts with Austrian patients, where a large proportion (40.1%) had no concern about payment at all. Differences were also observed between dabigatran and VKA sub-groups (see Table 1 of the supplementary material).

**Table 2:** Variation in treatment perception in Cohort B by country (overall, main analysis set)

PACQ-Q1 item	Country	Missing *N* (%)	Not at all *N* (%)	A little *N* (%)	Moderately *N* (%)	A lot *N* (%)	Extremely *N* (%)
A1: How confident are you that your anticoagulant therapy will prevent blood clots?	Austria	23 (6.7)	8 (2.3)	11 (3.2)	29 (8.5)	135 (39.5)	136 (39.8)
	Bulgaria	0 (0.0)	1 (1.0)	5 (4.9)	44 (43.1)	43 (42.2)	9 (8.8)
	Estonia	2 (2.0)	4 (3.9)	6 (5.9)	40 (39.2)	44 (43.1)	6 (5.9)
	Hungary	2 (5.7)	0 (0.0)	3 (8.6)	3 (8.6)	15 (42.9)	12 (34.3)
	Israel	0 (0.0)	0 (0.0)	2 (3.9)	25 (49.0)	17 (33.3)	7 (13.7)
	Latvia	2 (1.9)	3 (2.9)	8 (7.8)	24 (23.3)	57 (55.3)	9 (8.7)
	Poland	42 (3.1)	7 (0.5)	131 (9.6)	385 (28.4)	557 (41.0)	236 (17.4)
	Romania	47 (4.5)	4 (0.4)	48 (4.6)	313 (30.2)	487 (47.0)	137 (13.2)
	Russia	45 (2.3)	49 (2.5)	220 (11.2)	586 (29.7)	934 (47.4)	137 (7.0)
	Serbia	1 (0.4)	2 (0.9)	9 (3.9)	44 (19.2)	122 (53.3)	51 (22.3)
A2: Do you expect that your anticoagulant therapy will relieve some of the symptoms you experience?	Austria	23 (6.7)	38 (11.1)	84 (24.6)	82 (24.0)	91 (26.6)	24 (7.0)
	Bulgaria	0 (0.0)	6 (5.9)	32 (31.4)	38 (37.3)	22 (21.6)	4 (3.9)
	Estonia	2 (2.0)	11 (10.8)	13 (12.7)	30 (29.4)	35 (34.3)	11 (10.8)
	Hungary	2 (5.7)	6 (17.1)	8 (22.9)	9 (25.7)	7 (20.0)	3 (8.6)
	Israel	0 (0.0)	5 (9.8)	8 (15.7)	16 (31.4)	18 (35.3)	4 (7.8)
	Latvia	2 (1.9)	9 (8.7)	20 (19.4)	32 (31.1)	33 (32.0)	7 (6.8)
	Poland	42 (3.1)	72 (5.3)	275 (20.3)	409 (30.1)	437 (32.2)	123 (9.1)
	Romania	47 (4.5)	223 (21.5)	184 (17.8)	322 (31.1)	222 (21.4)	38 (3.7)
	Russia	45 (2.3)	231 (11.7)	410 (20.8)	590 (29.9)	570 (28.9)	125 (6.3)
	Serbia	1 (0.4)	18 (7.9)	54 (23.6)	69 (30.1)	72 (31.4)	15 (6.6)
A3: Do you expect that your anticoagulant therapy will cause side effects such as minor bruises or bleeding?	Austria	23 (6.7)	25 (7.3)	139 (40.6)	111 (32.5)	40 (11.7)	4 (1.2)
	Bulgaria	0 (0.0)	6 (5.9)	32 (31.4)	43 (42.2)	21 (20.6)	0 (0.0)
	Estonia	2 (2.0)	25 (24.5)	33 (32.4)	22 (21.6)	18 (17.6)	2 (2.0)
	Hungary	2 (5.7)	2 (5.7)	12 (34.3)	15 (42.9)	2 (5.7)	2 (5.7)
	Israel	0 (0.0)	10 (19.6)	10 (19.6)	21 (41.2)	8 (15.7)	2 (3.9)
	Latvia	2 (1.9)	11 (10.7)	40 (38.8)	43 (41.7)	5 (4.9)	2 (1.9)
	Poland	42 (3.1)	103 (7.6)	491 (36.2)	558 (41.1)	139 (10.2)	25 (1.8)
	Romania	47 (4.5)	205 (19.8)	414 (40.0)	310 (29.9)	58 (5.6)	2 (0.2)
	Russia	45 (2.3)	309 (15.7)	762 (38.7)	565 (28.7)	243 (12.3)	47 (2.4)
	Serbia	1 (0.4)	34 (14.8)	64 (27.9)	89 (38.9)	36 (15.7)	5 (2.2)
A4: How important is it for you to have an anticoagulant therapy that is easy to take?	Austria	23 (6.7)	8 (2.3)	13 (3.8)	24 (7.0)	105 (30.7)	169 (49.4)
	Bulgaria	0 (0.0)	0 (0.0)	1 (1.0)	22 (21.6)	48 (47.1)	31 (30.4)
	Estonia	2 (2.0)	2 (2.0)	4 (3.9)	18 (17.6)	44 (43.1)	32 (31.4)
	Hungary	2 (5.7)	0 (0.0)	0 (0.0)	2 (5.7)	17 (48.6)	14 (40.0)
	Israel	0 (0.0)	6 (11.8)	2 (3.9)	15 (29.4)	15 (29.4)	13 (25.5)
	Latvia	2 (1.9)	5 (4.9)	3 (2.9)	11 (10.7)	52 (50.5)	30 (29.1)
	Poland	42 (3.1)	6 (0.4)	56 (4.1)	167 (12.3)	838 (61.7)	249 (18.3)
	Romania	47 (4.5)	5 (0.5)	16 (1.5)	100 (9.7)	516 (49.8)	352 (34.0)
	Russia	45 (2.3)	60 (3.0)	126 (6.4)	360 (18.3)	1077 (54.6)	303 (15.4)
	Serbia	1 (0.4)	0 (0.0)	3 (1.3)	29 (12.7)	102 (44.5)	94 (41.0)
A5: How concerned are you about making mistakes when taking your anticoagulant therapy?	Austria	23 (6.7)	151 (44.2)	72 (21.1)	60 (17.5)	25 (7.3)	11 (3.2)
	Bulgaria	0 (0.0)	9 (8.8)	24 (23.5)	48 (47.1)	19 (18.6)	2 (2.0)
	Estonia	2 (2.0)	21 (20.6)	25 (24.5)	17 (16.7)	24 (23.5)	13 (12.7)
	Hungary	2 (5.7)	9 (25.7)	10 (28.6)	12 (34.3)	2 (5.7)	0 (0.0)
	Israel	0 (0.0)	13 (25.5)	8 (15.7)	21 (41.2)	7 (13.7)	2 (3.9)
	Latvia	2 (1.9)	8 (7.8)	25 (24.3)	27 (26.2)	37 (35.9)	4 (3.9)
	Poland	42 (3.1)	178 (13.1)	307 (22.6)	452 (33.3)	334 (24.6)	45 (3.3)
	Romania	47 (4.5)	77 (7.4)	190 (18.3)	255 (24.6)	337 (32.5)	130 (12.5)
	Russia	45 (2.3)	286 (14.5)	477 (24.2)	461 (23.4)	539 (27.3)	163 (8.3)
	Serbia	1 (0.4)	21 (9.2)	39 (17.0)	54 (23.6)	83 (36.2)	31 (13.5)
A6: How important is it for you to take care of your anticoagulant therapy by yourself?	Austria	23 (6.7)	6 (1.8)	19 (5.6)	37 (10.8)	71 (20.8)	186 (54.4)
	Bulgaria	0 (0.0)	1 (1.0)	2 (2.0)	29 (28.4)	49 (48.0)	21 (20.6)
	Estonia	2 (2.0)	3 (2.9)	2 (2.0)	16 (15.7)	53 (52.0)	26 (25.5)
	Hungary	2 (5.7)	0 (0.0)	0 (0.0)	2 (5.7)	25 (71.4)	6 (17.1)
	Israel	0 (0.0)	1 (2.0)	1 (2.0)	16 (31.4)	19 (37.3)	14 (27.5)
	Latvia	2 (1.9)	3 (2.9)	13 (12.6)	16 (15.5)	52 (50.5)	17 (16.5)
	Poland	42 (3.1)	14 (1.0)	66 (4.9)	199 (14.7)	793 (58.4)	244 (18.0)
	Romania	47 (4.5)	11 (1.1)	22 (2.1)	102 (9.8)	518 (50.0)	336 (32.4)
	Russia	45 (2.3)	68 (3.5)	155 (7.9)	411 (20.9)	1011 (51.3)	281 (14.3)
	Serbia	1 (0.4)	5 (2.2)	11 (4.8)	24 (10.5)	91 (39.7)	97 (42.4)
A7: How concerned are you about how much you may have to pay for your anticoagulant therapy?	Austria	23 (6.7)	137 (40.1)	73 (21.3)	62 (18.1)	33 (9.6)	14 (4.1)
	Bulgaria	0 (0.0)	4 (3.9)	6 (5.9)	48 (47.1)	32 (31.4)	12 (11.8)
	Estonia	2 (2.0)	13 (12.7)	10 (9.8)	24 (23.5)	23 (22.5)	30 (29.4)
	Hungary	2 (5.7)	5 (14.3)	12 (34.4)	11 (31.4)	3 (8.6)	2 (5.7)
	Israel	0 (0.0)	11 (21.6)	5 (9.8)	19 (37.3)	15 (29.4)	1 (2.0)
	Latvia	2 (1.9)	7 (6.8)	11 (10.7)	30 (29.1)	29 (28.2)	24 (23.3)
	Poland	42 (3.1)	120 (8.8)	195 (14.4)	414 (30.5)	435 (32.0)	152 (11.2)
	Romania	47 (4.5)	46 (4.4)	74 (7.1)	266 (25.7)	334 (32.2)	269 (26.0)
	Russia	45 (2.3)	201 (10.2)	257 (13.0)	519 (26.3)	614 (31.2)	335 (17.0)
	Serbia	1 (0.4)	26 (11.4)	35 (15.3)	53 (23.1)	76 (33.2)	38 (16.6)

Cohort B: patients newly initiated on dabigatran or a VKA

Data for Slovenia and the Czech Republic are not shown due to low patient numbers (*N* < 15)

PACT-Q, Perception of Anticoagulant Treatment Questionnaire; VKA, vitamin K antagonist

#### By age group

The variations of treatment expectations in different age groups measured by PACT-Q1 for Cohort B are summarized in Table 3. Overall, the distribution of answers to PACT-Q1 treatment expectation questions was similar between the three different age groups. Large proportions of patients in all age groups were confident that their anticoagulant treatment would prevent blood clots (39.4–50.0% answered “a lot”), found it important to have an anticoagulant treatment that was easy to take (52.6–52.9% answered “a lot”), and found it important for them to take care of their anticoagulant treatment by themselves (48.9–51.2% answered “a lot”). When asked about their confidence that the anticoagulant treatment would prevent blood clotting, a slightly higher proportion of patients in the < 65 years group (50.0%) answered “a lot” compared with the ≥ 75 years group (39.4%). Similar numbers across the three age groups expected their anticoagulant treatment to provide symptom relief (27.7–28.8% answered “a lot”, and 29.2–30.7% answered “moderately”), and expected their anticoagulant treatment to cause side effects (31.9–35.9% answered “moderately”, and 34.7–39.1% answered “a little”). Patient concerns about making mistakes when taking their medication ranged across all answers in all three age groups. Most patients answered “moderately” (26.6–27.6%) and “a lot” (27.6–30.9%) when asked about their concerns regarding payment for their treatment.

**Table 3:** Variation in treatment expectations at baseline by age group in Cohort B (overall, main analysis set)

PACT-Q1 item	Age group, years	Missing *N* (%)	Not at all *N* (%)	A little *N* (%)	Moderately *N* (%)	A lot *N* (%)	Extremely *N* (%)
A1: How confident are you that your anticoagulant therapy will prevent blood clots?	< 65	48 (2.7)	28 (1.6)	126 (7.1)	458 (25.9)	883 (50.0)	222 (12.6)
	65 to < 75	63 (3.2)	25 (1.3)	159 (8.1)	543 (27.7)	896 (45.8)	271 (13.8)
	≥ 75	56 (3.4)	25 (1.5)	160 (9.7)	503 (30.6)	647 (39.4)	252 (15.3)
A2: Do you expect that your anticoagulant therapy will relieve some of the symptoms you experience?	< 65	48 (2.7)	240 (13.6)	336 (19.0)	515 (29.2)	508 (28.8)	118 (6.7)
	65 to < 75	63 (3.2)	222 (11.3)	388 (19.8)	601 (30.7)	545 (27.8)	138 (7.1)
	≥ 75	56 (3.4)	160 (9.7)	371 (22.6)	498 (30.3)	457 (27.8)	101 (6.1)
A3: Do you expect that your anticoagulant therapy will cause side effects such as minor bruises or bleeding?	< 65	48 (2.7)	263 (14.9)	678 (38.4)	563 (31.9)	190 (10.8)	23 (1.3)
	65 to < 75	63 (3.2)	258 (13.2)	765 (39.1)	631 (32.2)	205 (10.5)	35 (1.8)
	≥ 75	56 (3.4)	216 (13.1)	570 (34.7)	590 (35.9)	178 (10.8)	33 (2.0)
A4: How important is it for you to have an anticoagulant therapy that is easy to take?	< 65	48 (2.7)	34 (1.9)	65 (3.7)	267 (15.1)	929 (52.6)	422 (23.9)
	65 to < 75	63 (3.2)	35 (1.8)	90 (4.6)	259 (13.2)	1031 (52.7)	479 (24.5)
	≥ 75	56 (3.4)	26 (1.6)	74 (4.5)	224 (13.6)	869 (52.9)	394 (24.0)
A5: How concerned are you about making mistakes when taking your anticoagulant therapy?	< 65	48 (2.7)	258 (14.6)	421 (23.9)	470 (26.6)	445 (25.2)	123 (7.0)
	65 to < 75	63 (3.2)	303 (15.5)	410 (21.0)	498 (25.4)	528 (27.0)	155 (7.9)
	≥ 75	56 (3.4)	222 (13.5)	358 (21.8)	445 (27.1)	438 (26.7)	124 (7.5)
A6: How important is it for you to take care of your anticoagulant therapy by yourself?	< 65	48 (2.7)	44 (2.5)	88 (5.0)	271 (15.4)	903 (51.2)	411 (23.3)
	65 to < 75	63 (3.2)	41 (2.1)	96 (4.9)	308 (15.7)	990 (50.6)	459 (23.5)
	≥ 75	56 (3.4)	31 (1.9)	112 (6.8)	275 (16.7)	803 (48.9)	366 (22.3)
A7: How concerned are you about how much you may have to pay for your anticoagulant therapy?	< 65	48 (2.7)	184 (10.4)	235 (13.3)	487 (27.6)	545 (30.9)	266 (15.1)
	65 to < 75	63 (3.2)	202 (10.3)	241 (12.3)	521 (26.6)	605 (30.9)	325 (16.6)
	≥ 75	56 (3.4)	195 (11.9)	205 (12.5)	445 (27.1)	454 (27.6)	288 (17.5)

Cohort B: patients newly initiated on dabigatran or a VKA

*PACT-Q* Perception of Anticoagulant Treatment Questionnaire, *VKA* vitamin K antagonist

When comparing the dabigatran and VKA sub-groups of Cohort B according to age group (see Tables 2, 3 and 4 of the supplementary material), a higher proportion of patients aged 65 to < 75 years in the dabigatran sub-group versus the VKA sub-group were extremely confident that their anticoagulant treatment would prevent blood clots (16.7% vs. 9.7% answered “extremely”, respectively) and for patients aged ≥ 75 years, 18.2% versus 11.1% answered “extremely”, respectively. Among patients aged < 65 years and 65 to < 75 years, more patients in the VKA sub-group versus the dabigatran sub-group were concerned about making mistakes when taking their medication (< 65 years, 31.8% vs. 23.0% answered “moderately”, respectively; but those aged 65 to < 75 years, 30.6% vs. 24.4% answered “a lot”, respectively).

## Discussion

This exploratory analysis was conducted as part of the observational RE-SONANCE study to investigate patient perceptions of anticoagulation treatment while receiving dabigatran or VKA for stroke prevention in AF in Europe (11 countries) and Israel. Perceptions were assessed by country and by age group. Assessment of treatment convenience and satisfaction (PACT-Q2) scores in different countries in Cohort A (Czech Republic, Hungary, Poland, Romania, and Russia) confirmed improved treatment satisfaction and convenience in favor of dabigatran compared with VKAs in patients who switched from VKA to dabigatran. Similarly, differences in PACT-Q2 scores between dabigatran and VKA sub-groups in Cohort B (Poland, Romania, and Russia) confirmed improved treatment satisfaction and convenience in favor of dabigatran compared with VKAs in patients newly initiated on either anticoagulant. Data from other countries could not be assessed due to low patient numbers. Changes in treatment convenience and satisfaction (PACT-Q2) scores were generally consistent across age groups and favored the dabigatran sub-group.

Assessment of treatment expectation (PACT-Q1) responses at baseline in Cohort B showed differences between countries. This reflects different perceptions of oral anticoagulation in patients with AF between different countries. This issue was also apparent in the IMPACT-AF trial [15]. Patients in Austria tended to be the most confident that their anticoagulant therapy would prevent blood clots. Patients in Estonia had the highest expectations regarding symptom relief, while a large proportion of patients in Romania had no expectations. Patients in Austria reported the least concern about making mistakes when taking their medication and, together with patients in Serbia, believed it was extremely important to take care of the anticoagulant therapy themselves. Extreme concerns regarding payment were expressed by patients in Estonia, Latvia, and Romania, while many Austrian patients had no concerns. The regional differences in treatment expectations across Europe highlighted in this analysis suggest the need for improvements in patient education regarding anticoagulation therapy. Assessment of treatment expectation (PACT-Q1) responses at baseline between different age groups in Cohort B revealed that overall, patients aged < 65 years tended to be more confident that their medication would prevent blood clotting compared with those aged ≥ 75 years. In the dabigatran sub-group versus the VKA sub-group, patients aged 65 to < 75 years and ≥ 75 years also tended to be more confident that their treatment would prevent blood clots.

This exploratory analysis of RE-SONANCE study data is subject to the limitations inherent to observational studies and exploratory analyses. The results were also influenced by low sample sizes. When assessing PACT-Q2 scores by country, changes in Austria, Bulgaria, Estonia, Israel, Latvia, Serbia, and Slovenia were statistically inconclusive. When assessing changes in PACT-Q1 scores in Cohort B, data could not be interpreted (Czech Republic, Hungary, and Slovenia) or should be interpreted with caution (Bulgaria, Estonia, Israel, and Latvia). Propensity score matching was conducted to control for channeling bias in Cohort B, but the model considered a limited number of comorbidities; those not included have the potential to cause residual confounding. Other potential sources of bias include reimbursement for anticoagulation therapy; patients may have been unwilling to express their true opinions in PACT-Q, or had difficulty in understanding the questions; those switching to dabigatran may have subconsciously considered the new therapy better than their previous therapy, particularly as the clinical reasons for switching from a VKA to dabigatran were not collected.

These limitations notwithstanding, data from this exploratory analysis support and expand upon published results from the RE-SONANCE study and resonate with other studies. When compared with the overall RE-SONANCE population, improvements in both treatment convenience and satisfaction (PACT-Q2) scores tended to be numerically higher in Hungary (Cohort A), Poland, and Romania (Cohorts A and B) [12]. Findings among the different age groups (< 65, 65 to < 75, and ≥ 75 years), however, were generally consistent with the overall population. Other than RE-SONANCE, few other studies have assessed patients’ perspectives of anticoagulant therapy in AF and, in particular, how these vary between countries and age groups [12]. A cross-sectional study conducted in France described real-world experience of direct OACs or VKAs for AF, and found that although patients in both groups had comparable QoL and adherence, those receiving a NOAC reported significantly greater treatment satisfaction (using PACT-Q2) [11]. QoL data from the phase III RE-LY^®^ trial, which included patients from 44 countries, revealed comparable EuroQol 5 dimensions (EQ-5D) utility and visual analog scores in the dabigatran and warfarin groups after 12 months of stable treatment [8,6]. The Prevention of Thromboembolic Events—European Registry in Atrial Fibrillation (PREFER in AF) Registry assessed QoL (EQ-5D-5 L) and satisfaction (PACT-Q2) in patients on stable treatment with a VKA, or those recently switched from a VKA to a NOAC [10]. Patients who had switched to NOACs from VKAs tended to be at lower risk than non-switchers and dissatisfied with VKA treatment. The need for increased patient awareness regarding OACs has been recognized previously [16, 17]. Therefore, as current guidelines for stroke prevention recommend patient involvement in treatment decisions, it will be important to increase patient confidence in dabigatran.

## Conclusions

This pre-specified, exploratory analysis from RE-SONANCE provides further confirmation of a preference toward switching to dabigatran from VKAs, or initiating dabigatran versus a VKA for stroke prevention in newly diagnosed patients with AF. This analysis highlights regional differences in treatment expectations across Europe.

## Supplementary Information

Below is the link to the electronic supplementary material.

Supplementary material 1 (DOCX 99.6 kb)

## Data Availability

To ensure independent interpretation of clinical study results, Boehringer Ingelheim grants all external authors access to all relevant material, including participant-level clinical study data, and relevant material as needed by them to fulfill their role and obligations as authors under the ICMJE criteria. Furthermore, clinical study documents (e.g. study report, study protocol, statistical analysis plan) and participant clinical study data are available to be shared after publication of the primary manuscript in a peer-reviewed journal and if regulatory activities are complete and other criteria met per the BI Policy on Transparency and Publication of Clinical Study Data: https://trials.boehringer-ingelheim.com/. Prior to providing access, documents will be examined, and, if necessary, redacted and the data will be de-identified, to protect the personal data of study participants and personnel, and to respect the boundaries of the informed consent of the study participants. Clinical Study Reports and Related Clinical Documents can also be requested via the link https://trials.boehringer-ingelheim.com/. All requests will be governed by a Document Sharing Agreement. Bona fide, qualified scientific and medical researchers may request access to de-identified, analysable participant clinical study data with corresponding documentation describing the structure and content of the datasets. Upon approval, and governed by a Data Sharing Agreement, data are shared in a secured data-access system for a limited period of 1 year, which may be extended upon request. Researchers should use the https://trials.boehringer-ingelheim.com/ link to request access to study data.
