# Supplementary material for: Patient perceptions of anticoagulant treatment with dabigatran or a vitamin K antagonist for stroke prevention in atrial fibrillation according to region and age: an exploratory analysis from the RE-SONANCE study
Source: J Thromb Thrombolysis. 2021 Apr 30;52(4):1195–206. doi: 10.1007/s11239-021-02450-2 (PMC8605976; doi:10.1007/s11239-021-02450-2)
Supplement: Supplementary file 1 — Supplementary material 1 (DOCX 99.6 kb) [file 11239_2021_2450_MOESM1_ESM.docx]

SUPPLEMENTARY MATERIAL

*Journal of Thrombosis and Thrombolysis*

Patient perceptions of anticoagulant treatment with dabigatran or a vitamin K antagonist for stroke prevention in atrial fibrillation according to region and age: an exploratory analysis from the RE-SONANCE study

Dragos Vinereanu^1^ · Dmitry Napalkov^2^ · Jutta Bergler-Klein^3^ · Bela Benczur^4^ · Martin Ciernik^5^ · Nina Gotcheva^6^ · Alexey Medvedchikov^5^ · Pentti Põder^7^ · Dragan Simić^8^ · Andris Skride^9^ · Wenbo Tang^10^ · Maria Trusz-Gluza^11^ · Jiří Vesely^12^

^1^ University of Medicine and Pharmacy Carol Davila, University and Emergency Hospital, Bucharest, Romania

^2^ I.M. Sechenov First Moscow State Medical University (Sechenov University), Moscow, Russian Federation

^3^ Department of Cardiology, University Clinic of Internal Medicine II, Medical University of Vienna, Vienna, Austria

^4^ Balassa Janos County Hospital, Szekszárd, Hungary

^5^ Boehringer Ingelheim RCV GmbH & Co. KG, Vienna, Austria

^6^ National Cardiology Hospital, Sofia, Bulgaria

^7^ North Estonia Medical Centre Foundation, Tallinn, Estonia

^8^ Clinic of Cardiology, Clinical Centre of Serbia, Faculty of Medicine, University of Belgrade, Belgrade, Serbia

^9^ Pauls Stradins Clinical University Hospital, and Riga Stradinš University, Riga, Latvia

^10^ Boehringer Ingelheim Pharmaceuticals, Inc., Ridgefield, CT, USA

^11^ Silesian Medical University, Katowice, Poland

^12^ Faculty of Medicine in Hradec Kralove, Charles University, and Edumed s.r.o., Broumov, Czech Republic

**Correspondence:** Dragos Vinereanu, MD, PhD, FESC, FRCP, Professor of Cardiology, University of Medicine and Pharmacy Carol Davila, University and Emergency Hospital, Bucharest, Splaiul Independentei 169, București 050078, Romania.

Telephone: +40 751 223 361. E-mail: [vinereanu@gmail.com](mailto:vinereanu@gmail.com)

**Table 1** Variation in treatment perception in Cohort B by country (by dabigatran or VKA sub-group, MAS)

| PACT-Q1 item | Country | Treatment group | Missing  *N* (%) | Not at all  *N* (%) | A little  *N* (%) | Moderately  *N* (%) | A lot  *N* (%) | Extremely  *N* (%) |
| --- | --- | --- | --- | --- | --- | --- | --- | --- |
| A1: How confident are you that your anticoagulant therapy will prevent blood clots? | Austria | Dabigatran | 15 (5.1) | 8 (2.7) | 10 (3.4) | 25 (8.5) | 112 (38.1) | 124 (42.2) |
|  |  | VKA | 8 (16.7) | 0 (0.0) | 1 (2.1) | 4 (8.3) | 23 (47.9) | 12 (25.0) |
|  | Bulgaria | Dabigatran | 0 (0.0) | 1 (1.6) | 3 (4.7) | 25 (39.1) | 29 (45.3) | 6 (9.4) |
|  |  | VKA | 0 (0.0) | 0 (0.0) | 2 (5.3) | 19 (50.0) | 14 (36.8) | 3 (7.9) |
|  | Estonia | Dabigatran | 2 (2.4) | 4 (4.8) | 5 (6.0) | 31 (36.9) | 38 (45.2) | 4 (4.8) |
|  |  | VKA | 0 (0.0) | 0 (0.0) | 1 (5.6) | 9 (50.0) | 6 (33.3) | 2 (11.1) |
|  | Hungary | Dabigatran | 2 (7.1) | 0 (0.0) | 2 (7.1) | 3 (10.7) | 14 (50.0) | 7 (25.0) |
|  |  | VKA | 0 (0.0) | 0 (0.0) | 1 (14.3) | 0 (0.0) | 1 (14.3) | 5 (71.4) |
|  | Israel | Dabigatran | 0 (0.0) | 0 (0.0) | 2 (3.9) | 25 (49.0) | 17 (33.3) | 7 (13.7) |
|  |  | VKA | 0 (0.0) | 0 (0.0) | 0 (0.0) | 0 (0.0) | 0 (0.0) | 0 (0.0) |
|  | Latvia | Dabigatran | 1 (1.6) | 1 (1.6) | 6 (9.8) | 11 (18.0) | 37 (60.7) | 5 (8.2) |
|  |  | VKA | 1 (2.4) | 2 (4.8) | 2 (4.8) | 13 (31.0) | 20 (47.6) | 4 (9.5) |
|  | Poland | Dabigatran | 32 (3.9) | 6 (0.7) | 75 (9.1) | 224 (27.1) | 335 (40.6) | 154 (18.6) |
|  |  | VKA | 10 (1.9) | 1 (0.2) | 56 (10.5) | 161 (30.3) | 222 (41.7) | 82 (15.4) |
|  | Romania | Dabigatran | 26 (4.6) | 3 (0.5) | 20 (3.5) | 135 (23.8) | 289 (51.0) | 94 (16.6) |
|  |  | VKA | 21 (4.5) | 1 (0.2) | 28 (6.0) | 178 (38.0) | 198 (42.2) | 43 (9.2) |
|  | Russia | Dabigatran | 23 (2.2) | 21 (2.0) | 101 (9.6) | 294 (27.8) | 537 (50.8) | 81 (7.7) |
|  |  | VKA | 22 (2.4) | 28 (3.1) | 119 (13.0) | 292 (31.9) | 397 (43.4) | 56 (6.1) |
|  | Serbia | Dabigatran | 1 (0.8) | 2 (1.6) | 5 (4.0) | 22 (17.5) | 64 (50.8) | 32 (25.4) |
|  |  | VKA | 0 (0.0) | 0 (0.0) | 4 (3.9) | 22 (21.4) | 58 (56.3) | 19 (18.4) |
| A2: Do you expect that your anticoagulant therapy will relieve some of the symptoms you experience? | Austria | Dabigatran | 15 (5.1) | 35 (11.9) | 75 (25.5) | 71 (24.1) | 75 (25.5) | 23 (7.8) |
|  |  | VKA | 8 (16.7) | 3 (6.3) | 9 (18.8) | 11 (22.9) | 16 (33.3) | 1 (2.1) |
|  | Bulgaria | Dabigatran | 0 (0.0) | 3 (4.7) | 17 (26.6) | 27 (42.2) | 15 (23.4) | 2 (3.1) |
|  |  | VKA | 0 (0.0) | 3 (7.9) | 15 (39.5) | 11 (28.9) | 7 (18.4) | 2 (5.3) |
|  | Estonia | Dabigatran | 2 (2.4) | 8 (9.5) | 11 (13.1) | 26 (31.0) | 28 (33.3) | 9 (10.7) |
|  |  | VKA | 0 (0.0) | 3 (16.7) | 2 (11.1) | 4 (22.2) | 7 (38.9) | 2 (11.1) |
|  | Hungary | Dabigatran | 2 (7.1) | 6 (21.4) | 8 (28.6) | 6 (21.4) | 4 (14.3) | 2 (7.1) |
|  |  | VKA | 0 (0.0) | 0 (0.0) | 0 (0.0) | 3 (42.9) | 3 (42.9) | 1 (14.3) |
|  | Israel | Dabigatran | 0 (0.0) | 5 (9.8) | 8 (15.7) | 16 (31.4) | 18 (35.3) | 4 (7.8) |
|  |  | VKA | 0 (0.0) | 0 (0.0) | 0 (0.0) | 0 (0.0) | 0 (0.0) | 0 (0.0) |
|  | Latvia | Dabigatran | 1 (1.6) | 5 (8.2) | 11 (18.0) | 22 (36.1) | 17 (27.9) | 5 (8.2) |
|  |  | VKA | 1 (2.4) | 4 (9.5) | 9 (21.4) | 10 (23.8) | 16 (38.1) | 2 (4.8) |
|  | Poland | Dabigatran | 32 (3.9) | 46 (5.6) | 154 (18.6) | 248 (30.0) | 257 (31.1) | 89 (10.8) |
|  |  | VKA | 10 (1.9) | 26 (4.9) | 121 (22.7) | 161 (30.3) | 180 (33.8) | 34 (6.4) |
|  | Romania | Dabigatran | 26 (4.6) | 118 (20.8) | 97 (17.1) | 176 (31.0) | 128 (22.6) | 22 (3.9) |
|  |  | VKA | 21 (4.5) | 205 (22.4) | 87 (18.6) | 146 (31.1) | 94 (20.0) | 16 (3.4) |
|  | Russia | Dabigatran | 23 (2.2) | 123 (11.6) | 196 (18.5) | 305 (28.9) | 327 (30.9) | 83 (7.9) |
|  |  | VKA | 22 (2.4) | 108 (11.8) | 214 (23.4) | 285 (31.2) | 243 (26.6) | 42 (4.6) |
|  | Serbia | Dabigatran | 1 (0.8) | 11 (8.7) | 24 (19.0) | 38 (30.2) | 41 (32.5) | 11 (8.7) |
|  |  | VKA | 0 (0.0) | 7 (6.8) | 30 (29.1) | 31 (30.1) | 31 (30.1) | 4 (3.9) |
| A3: Do you expect that your anticoagulant therapy will cause side effects such as minor bruises or bleeding? | Austria | Dabigatran | 15 (5.1) | 20 (6.8) | 127 (43.2) | 94 (32.0) | 34 (11.6) | 4 (1.4) |
|  |  | VKA | 8 (16.7) | 5 (10.4) | 12 (25.0) | 17 (35.4) | 6 (12.5) | 0 (0.0) |
|  | Bulgaria | Dabigatran | 0 (0.0) | 5 (7.8) | 24 (37.5) | 28 (43.8) | 7 (10.9) | 0 (0.0) |
|  |  | VKA | 0 (0.0) | 1 (2.6) | 8 (21.1) | 15 (39.5) | 14 (36.8) | 0 (0.0) |
|  | Estonia | Dabigatran | 2 (2.4) | 19 (22.6) | 27 (32.1) | 18 (21.4) | 16 (19.0) | 2 (2.4) |
|  |  | VKA | 0 (0.0) | 6 (33.3) | 6 (33.3) | 4 (22.2) | 2 (11.1) | 0 (0.0) |
|  | Hungary | Dabigatran | 2 (7.1) | 2 (7.1) | 11 (39.3) | 11 (39.3) | 0 (0.0) | 2 (7.1) |
|  |  | VKA | 0 (0.0) | 0 (0.0) | 1 (14.3) | 4 (57.1) | 2 (28.6) | 0 (0.0) |
|  | Israel | Dabigatran | 0 (0.0) | 10 (19.6) | 10 (19.6) | 21 (41.2) | 8 (15.7) | 2 (3.9) |
|  |  | VKA | 0 (0.0) | 0 (0.0) | 0 (0.0) | 0 (0.0) | 0 (0.0) | 0 (0.0) |
|  | Latvia | Dabigatran | 1 (1.6) | 7 (11.5) | 28 (45.9) | 21 (34.4) | 2 (3.3) | 2 (3.3) |
|  |  | VKA | 1 (2.4) | 4 (9.5) | 12 (28.6) | 22 (52.4) | 3 (7.1) | 0 (0.0) |
|  | Poland | Dabigatran | 32 (3.9) | 71 (8.6) | 296 (35.8) | 332 (40.2) | 78 (9.4) | 17 (2.1) |
|  |  | VKA | 10 (1.9) | 32 (6.0) | 195 (36.7) | 226 (42.5) | 61 (11.5) | 8 (1.5) |
|  | Romania | Dabigatran | 26 (4.6) | 137 (24.2) | 217 (38.3) | 155 (27.3) | 30 (5.3) | 2 (0.4) |
|  |  | VKA | 21 (4.5) | 68 (14.5) | 197 (42.0) | 155 (33.0) | 28 (6.0) | 0 (0.0) |
|  | Russia | Dabigatran | 23 (2.2) | 188 (17.8) | 406 (38.4) | 284 (26.9) | 133 (12.6) | 23 (2.2) |
|  |  | VKA | 22 (2.4) | 121 (13.2) | 356 (38.9) | 281 (30.7) | 110 (12.0) | 24 (2.6) |
|  | Serbia | Dabigatran | 1 (0.8) | 25 (19.8) | 44 (34.9) | 44 (34.9) | 10 (7.9) | 2 (1.6) |
|  |  | VKA | 0 (0.0) | 9 (8.7) | 20 (19.4) | 45 (43.7) | 26 (25.2) | 3 (2.9) |
| A4: How important is it for you to have an anticoagulant therapy that is easy to take? | Austria | Dabigatran | 15 (5.1) | 8 (2.7) | 12 (4.1) | 18 (6.1) | 89 (30.3) | 152 (51.7) |
|  |  | VKA | 8 (16.7) | 0 (0.0) | 1 (2.1) | 6 (12.5) | 16 (33.3) | 17 (35.4) |
|  | Bulgaria | Dabigatran | 0 (0.0) | 0 (0.0) | 1 (1.6) | 13 (20.3) | 30 (46.9) | 20 (31.3) |
|  |  | VKA | 0 (0.0) | 0 (0.0) | 0 (0.0) | 9 (23.7) | 18 (47.4) | 11 (28.9) |
|  | Estonia | Dabigatran | 2 (2.4) | 1 (1.2) | 4 (4.8) | 14 (16.7) | 37 (44.0) | 26 (31.0) |
|  |  | VKA | 0 (0.0) | 1 (5.6) | 0 (0.0) | 4 (22.2) | 7 (38.9) | 6 (33.3) |
|  | Hungary | Dabigatran | 2 (7.1) | 0 (0.0) | 0 (0.0) | 1 (3.6) | 16 (57.1) | 9 (32.1) |
|  |  | VKA | 0 (0.0) | 0 (0.0) | 0 (0.0) | 1 (14.3) | 1 (14.3) | 5 (71.4) |
|  | Israel | Dabigatran | 0 (0.0) | 6 (11.8) | 2 (3.9) | 15 (29.4) | 15 (29.4) | 13 (25.5) |
|  |  | VKA | 0 (0.0) | 0 (0.0) | 0 (0.0) | 0 (0.0) | 0 (0.0) | 0 (0.0) |
|  | Latvia | Dabigatran | 1 (1.6) | 4 (6.6) | 2 (3.3) | 3 (4.9) | 29 (47.5) | 22 (36.1) |
|  |  | VKA | 1 (2.4) | 1 (2.4) | 1 (2.4) | 8 (19.0) | 23 (54.8) | 8 (19.0) |
|  | Poland | Dabigatran | 32 (3.9) | 3 (0.4) | 20 (2.4) | 61 (7.4) | 532 (64.4) | 178 (21.5) |
|  |  | VKA | 10 (1.9) | 3 (0.6) | 36 (6.8) | 106 (19.9) | 306 (57.5) | 71 (13.3) |
|  | Romania | Dabigatran | 26 (4.6) | 5 (0.9) | 3 (0.5) | 26 (4.6) | 280 (49.4) | 227 (40.0) |
|  |  | VKA | 21 (4.5) | 0 (0.0) | 13 (2.8) | 74 (15.8) | 236 (50.3) | 125 (26.7) |
|  | Russia | Dabigatran | 23 (2.2) | 35 (3.3) | 59 (5.6) | 173 (16.4) | 593 (56.1) | 174 (16.5) |
|  |  | VKA | 22 (2.4) | 25 (2.7) | 67 (7.3) | 187 (20.5) | 484 (53.0) | 129 (14.1) |
|  | Serbia | Dabigatran | 1 (0.8) | 0 (0.0) | 1 (0.8) | 8 (6.3) | 46 (36.5) | 70 (55.6) |
|  |  | VKA | 0 (0.0) | 0 (0.0) | 2 (1.9) | 21 (20.4) | 56 (54.4) | 24 (23.3) |
| A5: How concerned are you about making mistakes when taking your anticoagulant therapy? | Austria | Dabigatran | 15 (5.1) | 139 (47.3) | 63 (21.4) | 47 (16.0) | 21 (7.1) | 9 (3.1) |
|  |  | VKA | 8 (16.7) | 12 (25.0) | 9 (18.8) | 13 (27.1) | 4 (8.3) | 2 (4.2) |
|  | Bulgaria | Dabigatran | 0 (0.0) | 6 (9.4) | 19 (29.7) | 31 (48.4) | 7 (10.9) | 1 (1.6) |
|  |  | VKA | 0 (0.0) | 3 (7.9) | 5 (13.2) | 17 (44.7) | 12 (31.6) | 1 (2.6) |
|  | Estonia | Dabigatran | 2 (2.4) | 15 (17.9) | 20 (23.8) | 14 (16.7) | 23 (27.4) | 10 (11.9) |
|  |  | VKA | 0 (0.0) | 6 (33.3) | 5 (27.8) | 3 (16.7) | 1 (5.6) | 3 (16.7) |
|  | Hungary | Dabigatran | 2 (7.1) | 5 (17.9) | 10 (35.7) | 9 (32.1) | 2 (7.1) | 0 (0.0) |
|  |  | VKA | 0 (0.0) | 4 (57.1) | 0 (0.0) | 3 (42.9) | 0 (0.0) | 0 (0.0) |
|  | Israel | Dabigatran | 0 (0.0) | 13 (25.5) | 8 (15.7) | 21 (41.2) | 7 (13.7) | 2 (3.9) |
|  |  | VKA | 0 (0.0) | 0 (0.0) | 0 (0.0) | 0 (0.0) | 0 (0.0) | 0 (0.0) |
|  | Latvia | Dabigatran | 1 (1.6) | 7 (11.5) | 16 (26.2) | 15 (24.6) | 20 (32.8) | 2 (3.3) |
|  |  | VKA | 1 (2.4) | 1 (2.4) | 9 (21.4) | 12 (28.6) | 17 (40.5) | 2 (4.8) |
|  | Poland | Dabigatran | 32 (3.9) | 128 (15.5) | 172 (20.8) | 261 (31.6) | 204 (24.7) | 29 (3.5) |
|  |  | VKA | 10 (1.9) | 50 (9.4) | 135 (25.4) | 191 (35.9) | 130 (24.4) | 16 (3.0) |
|  | Romania | Dabigatran | 26 (4.6) | 56 (9.9) | 93 (16.4) | 130 (22.9) | 189 (33.3) | 73 (12.9) |
|  |  | VKA | 21 (4.5) | 21 (4.5) | 97 (20.7) | 125 (26.7) | 148 (31.6) | 57 (12.2) |
|  | Russia | Dabigatran | 23 (2.2) | 183 (17.3) | 267 (25.3) | 209 (19.8) | 288 (27.2) | 87 (8.2) |
|  |  | VKA | 22 (2.4) | 103 (11.3) | 210 (23.0) | 252 (27.6) | 251 (27.5) | 76 (8.3) |
|  | Serbia | Dabigatran | 1 (0.8) | 16 (12.7) | 23 (18.3) | 28 (22.2) | 40 (31.7) | 18 (14.3) |
|  |  | VKA | 0 (0.0) | 5 (4.9) | 16 (15.5) | 26 (25.2) | 43 (41.7) | 13 (12.6) |
| A6: How important is it for you to take care of your anticoagulant therapy by yourself? | Austria | Dabigatran | 15 (5.1) | 6 (2.0) | 16 (5.4) | 32 (10.9) | 54 (18.4) | 171 (58.2) |
|  |  | VKA | 8 (16.7) | 0 (0.0) | 3 (6.3) | 5 (10.4) | 17 (35.4) | 15 (31.3) |
|  | Bulgaria | Dabigatran | 0 (0.0) | 1 (1.6) | 2 (3.1) | 20 (31.3) | 30 (46.9) | 11 (17.2) |
|  |  | VKA | 0 (0.0) | 0 (0.0) | 0 (0.0) | 9 (23.7) | 19 (50.0) | 10 (26.3) |
|  | Estonia | Dabigatran | 2 (2.4) | 3 (3.6) | 1 (1.2) | 13 (15.5) | 44 (52.4) | 21 (25.0) |
|  |  | VKA | 0 (0.0) | 0 (0.0) | 1 (5.6) | 3 (16.7) | 9 (50.0) | 5 (27.8) |
|  | Hungary | Dabigatran | 2 (7.1) | 0 (0.0) | 0 (0.0) | 0 (0.0) | 22 (78.6) | 4 (14.3) |
|  |  | VKA | 0 (0.0) | 0 (0.0) | 0 (0.0) | 2 (28.6) | 3 (42.9) | 2 (28.6) |
|  | Israel | Dabigatran | 0 (0.0) | 1 (2.0) | 1 (2.0) | 16 (31.4) | 19 (37.3) | 14 (27.5) |
|  |  | VKA | 0 (0.0) | 0 (0.0) | 0 (0.0) | 0 (0.0) | 0 (0.0) | 0 (0.0) |
|  | Latvia | Dabigatran | 1 (1.6) | 2 (3.3) | 7 (11.5) | 9 (14.8) | 30 (49.2) | 12 (19.7) |
|  |  | VKA | 1 (2.4) | 1 (2.4) | 6 (14.3) | 7 (16.7) | 22 (52.4) | 5 (11.9) |
|  | Poland | Dabigatran | 32 (3.9) | 5 (0.6) | 32 (3.9) | 80 (9.7) | 503 (60.9) | 174 (21.1) |
|  |  | VKA | 10 (1.9) | 9 (1.7) | 34 (6.4) | 119 (22.4) | 290 (54.5) | 70 (13.2) |
|  | Romania | Dabigatran | 26 (4.6) | 6 (1.1) | 7 (1.2) | 34 (6.0) | 291 (51.3) | 203 (35.8) |
|  |  | VKA | 21 (4.5) | 5 (1.1) | 15 (3.2) | 68 (14.5) | 227 (48.4) | 133 (28.4) |
|  | Russia | Dabigatran | 23 (2.2) | 39 (3.7) | 84 (7.9) | 183 (17.3) | 556 (52.6) | 172 (16.3) |
|  |  | VKA | 22 (2.4) | 29 (3.2) | 71 (7.8) | 228 (24.9) | 455 (49.8) | 109 (11.9) |
|  | Serbia | Dabigatran | 1 (0.8) | 4 (3.2) | 5 (4.0) | 10 (7.9) | 39 (31.0) | 67 (53.2) |
|  |  | VKA | 0 (0.0) | 1 (1.0) | 6 (5.8) | 14 (13.6) | 52 (50.5) | 30 (29.1) |
| A7: How concerned are you about how much you may have to pay for your anticoagulant therapy? | Austria | Dabigatran | 15 (5.1) | 124 (42.2) | 71 (24.1) | 50 (17.0) | 20 (6.8) | 14 (4.8) |
|  |  | VKA | 8 (16.7) | 13 (27.1) | 2 (4.2) | 12 (25.0) | 13 (27.1) | 0 (0.0) |
|  | Bulgaria | Dabigatran | 0 (0.0) | 2 (3.1) | 5 (7.8) | 33 (51.6) | 18 (28.1) | 6 (9.4) |
|  |  | VKA | 0 (0.0) | 2 (5.3) | 1 (2.6) | 15 (39.5) | 14 (36.8) | 6 (15.8) |
|  | Estonia | Dabigatran | 2 (2.4) | 10 (11.9) | 10 (11.9) | 22 (26.2) | 20 (23.8) | 20 (23.8) |
|  |  | VKA | 0 (0.0) | 3 (16.7) | 0 (0.0) | 2 (11.1) | 3 (16.7) | 10 (55.6) |
|  | Hungary | Dabigatran | 2 (7.1) | 3 (10.7) | 12 (42.9) | 8 (28.6) | 1 (3.6) | 2 (7.1) |
|  |  | VKA | 0 (0.0) | 2 (28.6) | 0 (0.0) | 3 (42.9) | 2 (28.6) | 0 (0.0) |
|  | Israel | Dabigatran | 0 (0.0) | 11 (21.6) | 5 (9.8) | 19 (37.3) | 15 (29.4) | 1 (2.0) |
|  |  | VKA | 0 (0.0) | 0 (0.0) | 0 (0.0) | 0 (0.0) | 0 (0.0) | 0 (0.0) |
|  | Latvia | Dabigatran | 1 (1.6) | 6 (9.8) | 8 (13.1) | 19 (31.1) | 16 (26.2) | 11 (18.0) |
|  |  | VKA | 1 (2.4) | 1 (2.4) | 3 (7.1) | 11 (26.2) | 13 (31.0) | 13 (31.0) |
|  | Poland | Dabigatran | 32 (3.9) | 64 (7.7) | 148 (17.9) | 302 (36.6) | 224 (27.1) | 56 (6.8) |
|  |  | VKA | 10 (1.9) | 56 (10.5) | 47 (8.8) | 112 (21.1) | 211 (39.7) | 96 (18.0) |
|  | Romania | Dabigatran | 26 (4.6) | 32 (5.6) | 53 (9.3) | 229 (40.4) | 150 (26.5) | 77 (13.6) |
|  |  | VKA | 21 (4.5) | 14 (3.0) | 21 (4.5) | 37 (7.9) | 184 (39.2) | 192 (40.9) |
|  | Russia | Dabigatran | 23 (2.2) | 121 (11.4) | 156 (14.8) | 328 (31.0) | 277 (26.2) | 152 (14.4) |
|  |  | VKA | 22 (2.4) | 80 (8.8) | 101 (11.1) | 191 (20.9) | 337 (36.9) | 183 (20.0) |
|  | Serbia | Dabigatran | 1 (0.8) | 18 (14.3) | 25 (19.8) | 43 (34.1) | 34 (27.0) | 5 (4.0) |
|  |  | VKA | 0 (0.0) | 8 (7.8) | 10 (9.7) | 10 (9.7) | 42 (40.8) | 33 (32.0) |

Cohort B: patients newly initiated on dabigatran or a VKA.

Data for Slovenia and the Czech Republic are not shown due to low patient numbers (*N* < 15).

MAS, main analysis set; PACT-Q, Perception of Anticoagulant Treatment Questionnaire; VKA, vitamin K antagonist.

**Table 2** Variation in treatment expectations at baseline in patients aged < 65 years in Cohort B (MAS)

| PACT-Q1 item | Treatment group | Missing  *N* (%) | Not at all  *N* (%) | A little  *N* (%) | Moderately  *N* (%) | A lot  *N* (%) | Extremely  *N* (%) |
| --- | --- | --- | --- | --- | --- | --- | --- |
| A1: How confident are you that your anticoagulant therapy will prevent blood clots? | Dabigatran | 29 (2.8) | 16 (1.5) | 67 (6.4) | 247 (23.7) | 538 (51.6) | 145 (13.9) |
|  | VKA | 19 (2.6) | 12 (1.7) | 59 (8.2) | 211 (29.2) | 345 (47.7) | 77 (10.7) |
|  | Overall | 48 (2.7) | 28 (1.6) | 126 (7.1) | 458 (25.9) | 883 (50.0) | 222 (12.6) |
| A2: Do you expect that your anticoagulant therapy will relieve some of the symptoms you experience? | Dabigatran | 29 (2.8) | 142 (13.6) | 182 (17.5) | 301 (28.9) | 305 (29.3) | 83 (8.0) |
|  | VKA | 19 (2.6) | 98 (13.6) | 154 (21.3) | 214 (29.6) | 203 (28.1) | 35 (4.8) |
|  | Overall | 48 (2.7) | 240 (13.6) | 336 (19.0) | 515 (29.2) | 508 (28.8) | 118 (6.7) |
| A3: Do you expect that your anticoagulant therapy will cause side effects such as minor bruises or bleeding? | Dabigatran | 29 (2.8) | 181 (17.4) | 403 (38.7) | 314 (30.1) | 102 (9.8) | 13 (1.2) |
|  | VKA | 19 (2.6) | 82 (11.3) | 275 (38.0) | 249 (34.4) | 88 (12.2) | 10 (1.4) |
|  | Overall | 48 (2.7) | 263 (14.9) | 678 (38.4) | 563 (31.9) | 190 (10.8) | 23 (1.3) |
| A4: How important is it for you to have an anticoagulant therapy that is easy to take? | Dabigatran | 29 (2.8) | 19 (1.8) | 37 (3.6) | 116 (11.1) | 550 (52.8) | 291 (27.9) |
|  | VKA | 19 (2.6) | 15 (2.1) | 28 (3.9) | 151 (20.9) | 379 (52.4) | 131 (18.1) |
|  | Overall | 48 (2.7) | 34 (1.9) | 65 (3.7) | 267 (15.1) | 929 (52.6) | 422 (23.9) |
| A5: How concerned are you about making mistakes when taking your anticoagulant therapy? | Dabigatran | 29 (2.8) | 185 (17.8) | 250 (24.0) | 240 (23.0) | 269 (25.8) | 69 (6.6) |
|  | VKA | 19 (2.6) | 73 (10.1) | 171 (23.7) | 230 (31.8) | 176 (24.3) | 54 (7.5) |
|  | Overall | 48 (2.7) | 258 (14.6) | 421 (23.9) | 470 (26.6) | 445 (25.2) | 123 (7.0) |
| A6: How important is it for you to take care of your anticoagulant therapy by yourself? | Dabigatran | 29 (2.8) | 27 (2.6) | 52 (5.0) | 135 (13.0) | 532 (51.1) | 267 (25.6) |
|  | VKA | 19 (2.6) | 17 (2.4) | 36 (5.0) | 136 (18.8) | 371 (51.3) | 144 (19.9) |
|  | Overall | 48 (2.7) | 44 (2.5) | 88 (5.0) | 271 (15.4) | 903 (51.2) | 411 (23.3) |
| A7: How concerned are you about how much you may have to pay for your anticoagulant therapy? | Dabigatran | 29 (2.8) | 130 (12.5) | 167 (16.0) | 356 (34.2) | 250 (24.0) | 110 (10.6) |
|  | VKA | 19 (2.6) | 54 (7.5) | 68 (9.4) | 131 (18.1) | 295 (40.8) | 156 (21.6) |
|  | Overall | 48 (2.7) | 184 (10.4) | 235 (13.3) | 487 (27.6) | 545 (30.9) | 266 (15.1) |

Cohort B: patients newly initiated on dabigatran or a VKA.

MAS, main analysis set; PACT-Q, Perception of Anticoagulant Treatment Questionnaire; VKA, vitamin K antagonist.

**Table 3** Variation in treatment expectations at baseline in patients aged 65 to < 75 years in Cohort B (MAS)

| PACT-Q1 item | Treatment group | Missing  *N* (%) | Not at all  *N* (%) | A little  *N* (%) | Moderately  *N* (%) | A lot  *N* (%) | Extremely  *N* (%) |
| --- | --- | --- | --- | --- | --- | --- | --- |
| A1: How confident are you that your anticoagulant therapy will prevent blood clots? | Dabigatran | 38 (3.3) | 18 (1.6) | 79 (6.8) | 274 (23.7) | 552 (47.8) | 193 (16.7) |
|  | VKA | 25 (3.1) | 7 (0.9) | 80 (10.0) | 269 (33.5) | 344 (42.8) | 78 (9.7) |
|  | Overall | 63 (3.2) | 25 (1.3) | 159 (8.1) | 543 (27.7) | 896 (45.8) | 271 (13.8) |
| A2: Do you expect that your anticoagulant therapy will relieve some of the symptoms you experience? | Dabigatran | 38 (3.3) | 128 (11.1) | 216 (18.7) | 352 (30.5) | 322 (27.9) | 98 (8.5) |
|  | VKA | 25 (3.1) | 94 (11.7) | 172 (21.4) | 249 (31.0) | 223 (27.8) | 40 (5.0) |
|  | Overall | 63 (3.2) | 222 (11.3) | 388 (19.8) | 601 (30.7) | 545 (27.8) | 138 (7.1) |
| A3: Do you expect that your anticoagulant therapy will cause side effects such as minor bruises or bleeding? | Dabigatran | 38 (3.3) | 167 (14.5) | 446 (38.6) | 369 (32.0) | 112 (9.7) | 22 (1.9) |
|  | VKA | 25 (3.1) | 91 (11.3) | 319 (39.7) | 262 (32.6) | 93 (11.6) | 13 (1.6) |
|  | Overall | 63 (3.2) | 258 (13.2) | 765 (39.1) | 631 (32.2) | 205 (10.5) | 35 (1.8) |
| A4: How important is it for you to have an anticoagulant therapy that is easy to take? | Dabigatran | 38 (3.3) | 29 (2.5) | 43 (3.7) | 106 (9.2) | 602 (52.2) | 336 (29.1) |
|  | VKA | 25 (3.1) | 6 (0.7) | 47 (5.9) | 153 (19.1) | 429 (53.4) | 143 (17.8) |
|  | Overall | 63 (3.2) | 35 (1.8) | 90 (4.6) | 259 (13.2) | 1031 (52.7) | 479 (24.5) |
| A5: How concerned are you about making mistakes when taking your anticoagulant therapy? | Dabigatran | 38 (3.3) | 226 (19.6) | 233 (20.2) | 278 (24.1) | 282 (24.4) | 97 (8.4) |
|  | VKA | 25 (3.1) | 77 (9.6) | 177 (22.0) | 220 (27.4) | 246 (30.6) | 58 (7.2) |
|  | Overall | 63 (3.2) | 303 (15.5) | 410 (21.0) | 498 (25.4) | 528 (27.0) | 155 (7.9) |
| A6: How important is it for you to take care of your anticoagulant therapy by yourself? | Dabigatran | 38 (3.3) | 23 (2.0) | 52 (4.5) | 136 (11.8) | 569 (49.3) | 336 (29.1) |
|  | VKA | 25 (3.1) | 18 (2.2) | 44 (5.5) | 172 (21.4) | 421 (52.4) | 123 (15.3) |
|  | Overall | 63 (3.2) | 41 (2.1) | 96 (4.9) | 308 (15.7) | 990 (50.6) | 459 (23.5) |
| A7: How concerned are you about how much you may have to pay for your anticoagulant therapy? | Dabigatran | 38 (3.3) | 139 (12.0) | 172 (14.9) | 373 (32.3) | 309 (26.8) | 123 (10.7) |
|  | VKA | 25 (3.1) | 63 (7.8) | 69 (8.6) | 148 (18.4) | 296 (36.9) | 202 (25.2) |
|  | Overall | 63 (3.2) | 202 (10.3) | 241 (12.3) | 521 (26.6) | 605 (30.9) | 325 (16.6) |

Cohort B: patients newly initiated on dabigatran or a VKA.

MAS, main analysis set; PACT-Q, Perception of Anticoagulant Treatment Questionnaire; VKA, vitamin K antagonist.

**Table 4** Variation in treatment expectations at baseline in patients aged ≥ 75 years in Cohort B (MAS)

| PACT-Q1 item | Treatment group | Missing  *N* (%) | Not at all  *N* (%) | A little  *N* (%) | Moderately  *N* (%) | A lot  *N* (%) | Extremely  *N* (%) |
| --- | --- | --- | --- | --- | --- | --- | --- |
| A1: How confident are you that your anticoagulant therapy will prevent blood clots? | Dabigatran | 36 (3.7) | 12 (1.2) | 84 (8.5) | 282 (28.7) | 390 (39.7) | 179 (18.2) |
|  | VKA | 20 (3.0) | 13 (2.0) | 76 (11.5) | 221 (33.5) | 257 (38.9) | 73 (11.1) |
|  | Overall | 56 (3.4) | 25 (1.5) | 160 (9.7) | 503 (30.6) | 647 (39.4) | 252 (15.3) |
| A2: Do you expect that your anticoagulant therapy will relieve some of the symptoms you experience? | Dabigatran | 36 (3.7) | 91 (9.3) | 209 (21.3) | 291 (29.6) | 284 (28.9) | 72 (7.3) |
|  | VKA | 20 (3.0) | 69 (10.5) | 162 (24.5) | 207 (31.4) | 173 (26.2) | 29 (4.4) |
|  | Overall | 56 (3.4) | 160 (9.7) | 371 (22.6) | 498 (30.3) | 457 (27.8) | 101 (6.1) |
| A3: Do you expect that your anticoagulant therapy will cause side effects such as minor bruises or bleeding? | Dabigatran | 36 (3.7) | 139 (14.1) | 352 (35.8) | 329 (33.5) | 106 (10.8) | 21 (2.1) |
|  | VKA | 20 (3.0) | 77 (11.7) | 218 (33.0) | 261 (39.5) | 72 (10.9) | 12 (1.8) |
|  | Overall | 56 (3.4) | 216 (13.1) | 570 (34.7) | 590 (35.9) | 178 (10.8) | 33 (2.0) |
| A4: How important is it for you to have an anticoagulant therapy that is easy to take? | Dabigatran | 36 (3.7) | 15 (1.5) | 26 (2.6) | 111 (11.3) | 524 (53.3) | 271 (27.6) |
|  | VKA | 20 (3.0) | 11 (1.7) | 48 (7.3) | 113 (17.1) | 345 (52.3) | 123 (18.6) |
|  | Overall | 56 (3.4) | 26 (1.6) | 74 (4.5) | 224 (13.6) | 869 (52.9) | 394 (24.0) |
| A5: How concerned are you about making mistakes when taking your anticoagulant therapy? | Dabigatran | 36 (3.7) | 164 (16.7) | 215 (21.9) | 249 (25.3) | 253 (25.7) | 66 (6.7) |
|  | VKA | 20 (3.0) | 58 (8.8) | 143 (21.7) | 196 (29.7) | 185 (28.0) | 58 (8.8) |
|  | Overall | 56 (3.4) | 222 (13.5) | 358 (21.8) | 445 (27.1) | 438 (26.7) | 124 (7.5) |
| A6: How important is it for you to take care of your anticoagulant therapy by yourself? | Dabigatran | 36 (3.7) | 18 (1.8) | 53 (5.4) | 127 (12.9) | 498 (50.7) | 251 (25.5) |
|  | VKA | 20 (3.0) | 13 (2.0) | 59 (8.9) | 148 (22.4) | 305 (46.2) | 115 (17.4) |
|  | Overall | 56 (3.4) | 31 (1.9) | 112 (6.8) | 275 (16.7) | 803 (48.9) | 366 (22.3) |
| A7: How concerned are you about how much you may have to pay for your anticoagulant therapy? | Dabigatran | 36 (3.7) | 129 (13.1) | 156 (15.9) | 328 (33.4) | 221 (22.5) | 113 (11.5) |
|  | VKA | 20 (3.0) | 66 (10.0) | 49 (7.4) | 117 (17.7) | 233 (35.3) | 175 (26.5) |
|  | Overall | 56 (3.4) | 195 (11.9) | 205 (12.5) | 445 (27.1) | 454 (27.6) | 288 (17.5) |

Cohort B: patients newly initiated on dabigatran or a VKA.

MAS, main analysis set; PACT-Q, Perception of Anticoagulant Treatment Questionnaire; VKA, vitamin K antagonist.
